# Optical Coherence Tomography Imaging of the Lamina Cribrosa: Structural Biomarkers in Nonglaucomatous Diseases

**DOI:** 10.1155/2021/8844614

**Published:** 2021-02-19

**Authors:** Alice Paulo, Pedro G. Vaz, Danilo Andrade De Jesus, Luisa Sánchez Brea, Jan Van Eijgen, João Cardoso, Theo van Walsum, Stefan Klein, Ingeborg Stalmans, João Barbosa Breda

**Affiliations:** ^1^Laboratory for Instrumentation, Biomedical Engineering and Radiation Physics (LIBPhys-UC), Department of Physics, University of Coimbra, Coimbra, Portugal; ^2^Biomedical Imaging Group Rotterdam, Department of Radiology & Nuclear Medicine, Erasmus MC, Rotterdam, Netherlands; ^3^Research Group Ophthalmology, Department of Neurosciences, KU Leuven, Leuven, Belgium; ^4^Department of Ophthalmology, University Hospitals UZ Leuven, Leuven, Belgium; ^5^Cardiovascular R&D Center, Faculty of Medicine of the University of Porto, Porto, Portugal; ^6^Ophthalmology Department, Centro Hospitalar e Universitário São João, Porto, Portugal

## Abstract

The lamina cribrosa (LC) is an active structure that responds to the strain by changing its morphology. Abnormal changes in LC morphology are usually associated with, and indicative of, certain pathologies such as glaucoma, intraocular hypertension, and myopia. Recent developments in optical coherence tomography (OCT) have enabled detailed *in vivo* studies about the architectural characteristics of the LC. Structural characteristics of the LC have been widely explored in glaucoma management. However, information about which LC biomarkers could be useful for the diagnosis, and follow-up, of other diseases besides glaucoma is scarce. Hence, this literature review aims to summarize the role of the LC in nonophthalmic and ophthalmic diseases other than glaucoma. PubMed was used to perform a systematic review on the LC features that can be extracted from OCT images. All imaging features are presented and discussed in terms of their importance and applicability in clinical practice. A total of 56 studies were included in this review. Overall, LC depth (LCD) and thickness (LCT) have been the most studied features, appearing in 75% and 45% of the included studies, respectively. These biomarkers were followed by the prelaminar tissue thickness (21%), LC curvature index (5.4%), LC global shape index (3.6%), LC defects (3.6%), and LC strains/deformations (1.8%). Overall, the disease groups showed a thinner LC (smaller LCT) and a deeper ONH cup (larger LCD), with some exceptions. A large variability between approaches used to compute LC biomarkers has been observed, highlighting the importance of having automated and standardized methodologies in LC analysis. Moreover, further studies are needed to identify the pathologies where LC features have a diagnostic and/or prognostic value.

## 1. Introduction

The lamina cribrosa (LC) is a mesh-like structure localized in the posterior scleral canal of the optic nerve head (ONH), allowing retinal ganglion cell (RGC) axons to pass through to the brain. It is a fenestrated complex that also accommodates vessels that nourish the retina. A large circumpapillary ring of collagen and elastin fibers, in the immediate peripapillary sclera, protects the LC against the mechanical strain, such as that induced by an imbalance between intraocular pressure (IOP) and intracranial pressure (ICP) [1, 2]. Due to its anatomical location, between two differently pressurized compartments, there is a pressure gradient along the LC, denominated translaminar pressure difference (TLPD), which can be calculated as the difference between IOP and ICP in the subarachnoid space (SAS) [3, 4]. Despite being an extremely relevant structure to the eye's anatomy and function, little is known about the LC. LC morphology plays an important role in the development and progression of ophthalmic pathologies, notably on glaucomatous optic neuropathy, intraocular hypertension, and myopia [5–8]. The structural deformation and the correlated compression across the LC lead to blockade of axonal transport and eventually RGC death [9].

**Figure 1:**
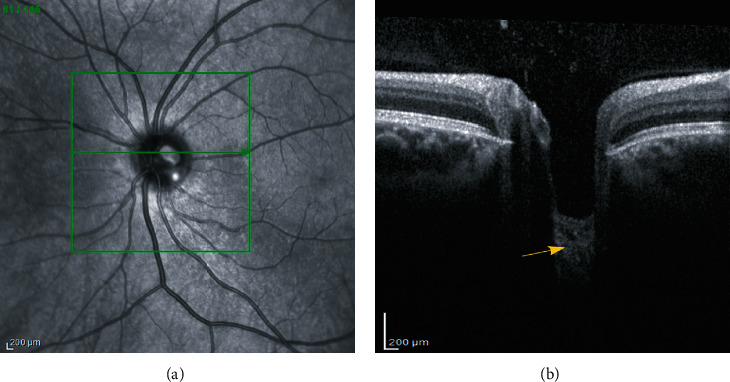
Retinal fundus photograph (a) and EDI-OCT B-scan at the optic nerve head (b). The green line denotes the location of the B-scan in the fundus image. The yellow arrow points to the lamina cribrosa region.

Recent advances in *in vivo* medical imaging techniques, such as optical coherence tomography (OCT), have allowed the visualization of deep connective tissues, including the LC, in greater detail (Figure 1) [10, 11].

Specific developments in OCT software, such as enhanced depth imaging (EDI), and light-attenuation correction software such as adaptive compensation (AC) significantly improved the visibility of the LC without compromising acquisition time. EDI-OCT was originally developed in order to improve the visualization of the choroid, although it has also been adopted to improve cross-sectional images of the LC. AC is a postprocessing technique developed to remove blood vessel shadows and enhance tissue contrast in order to facilitate posterior LC surface detection [12, 13]. In addition to these software developments, several studies have shown that swept-source OCT (SS-OCT) further improves the visualization of the LC [14, 15].

While an increasing number of works have studied the relevance of the LC (and its changes) in glaucoma [16], data on other diseases are still scarce. Hence, this review intends to provide a broader vision and a better comprehension of the measurable laminar structural features that have been identified as relevant for nonglaucomatous pathologies in the published literature.

## 2. Methods

### 2.1. Study Selection

**Figure 2:**
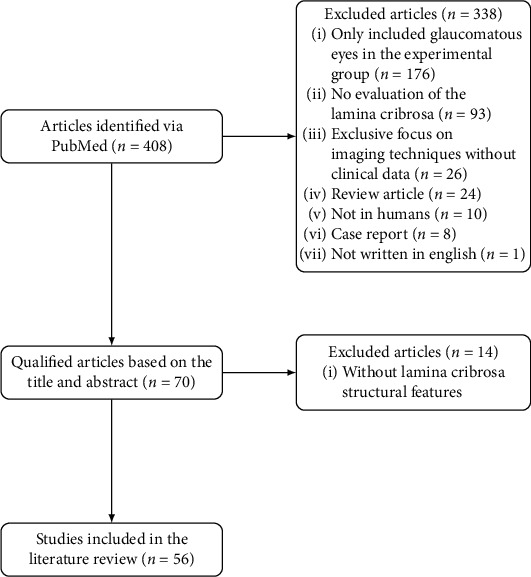
Flowchart of the collected data.

A literature search was conducted in the MEDLINE (PubMed) bibliographic database on 15th May 2020. The search query was (optical coherence tomography NOT angiography) AND (lamina OR cribrosa). Only articles published in English were considered, and no publication date restriction was added. The exclusion criteria were (i) only included glaucomatous eyes in the experimental group; (ii) not conducted in humans; (iii) review articles or case reports; (iv) exclusive focus on imaging techniques and not presenting clinical data; (v) no evaluation of the lamina cribrosa; and (vi) no mention to LC structural parameters and how they were measured/extracted. This led to a total of 408 references, which were narrowed down to 56 after title/abstract screening, followed by a full-text screening (see Figure 2). The 56 included studies provided quantitative values for each of the analyzed features and described how the quantification was performed.

### 2.2. Data Collection

In this review, our main aim was to identify potential biomarkers in the morphology of the LC that were associated with, and indicative of, certain pathologies. Therefore, we have opted to only report those that performed a statistical comparison between an experimental and a control group. The extracted data for each paper consist of the LC structural parameters, their mean and standard deviation (SD), and the *p* values of the statistical analysis performed between experimental and control groups. Moreover, the image processing methodology applied to compute each feature was taken into account for posterior comparisons.

For all the included articles, the following characteristics are obtained and presented in Table 1: sample size, including the number of patients and eyes per group; age and statistical comparison (*p* value) between control and experimental groups; OCT device model, manufacturer, and light-source wavelength; cutoff value for the signal strength index (SSI) or similar qualitative image criteria used to exclude patients/eyes; and field of view of the OCT image [17]. Moreover, the procedures followed to measure the LC features, and their respective values, were also collected and compiled.

Data collection comprehended all structural components related to the LC and the surrounding ONH region that were included on the OCT B-scan images. Several locations, planes (superior, middle, and inferior), and sectors were considered for the measurements. The sectors were defined according to the Garway-Heath map [71]. The approach of data extraction by one investigator (ASP) with further verification by a senior author (JBB) has been used, as this has been demonstrated to be as accurate as double independent data extraction [72].

### 2.3. Data Analysis

The obtained data were used to calculate the frequency of each LC structural feature in the published literature and to determine the mean values of the most frequently reported features. Statistical relevance, given by the *p* value, was also taken as a complement for study results and to comprehend the relation and differences found between study groups. To average the data for each LC parameter, pooled mean and pooled SD were determined according to equations (1) and (2), respectively, where *N* represents the number of eyes included in the study, *M* the mean value, SD the standard deviation, and *n* the number of studies analyzed:

(1) $$\begin{array}{c} \text{pooled mean}=\frac{\sum_{i=1}^{n}N_{i}\times M_{i}}{\sum_{i=1}^{n}N_{i}}, \end{array}$$

(2) $$\begin{array}{c} \text{pooled SD}=\sqrt{\frac{\sum_{i=1}^{n}\left(N_{i}-1\right)\times {\text{SD}}_{i}^{2}}{\sum_{i=1}^{n}\left(N_{i}-1\right)}}. \end{array}$$

## 3. Results

All structural LC features were analyzed, and the studies were organized in three groups: healthy group (*n* = 19), nonophthalmic disease group (*n* = 6), and ophthalmic (nonglaucomatous) disease group (*n* = 31). Overall, LC depth (LCD) and LC thickness (LCT) have been the most studied features, appearing in 75% and 44.6% from the total articles. Other features, such as prelaminar tissue thickness (PTT) (21.4%) studied in ophthalmic and nonophthalmic diseases, and lamina cribrosa curvature index (LCCI) (5.4%), LC global shape index (3.6%), LC defects (3.6%), slope of the LC (3.6%), distance between the inner surface of the LC (3.6%), SAS (3.6%), and LC strains/deformations (1.8%) studied in ophthalmic diseases only, are also referenced but in fewer studies.

**Figure 3:**
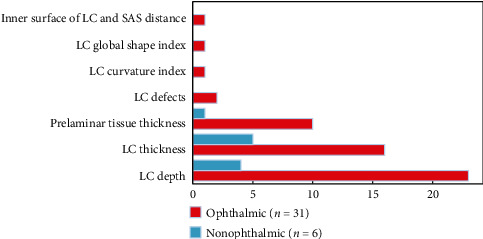
Number of occurrences of each lamina cribrosa structural feature published in the literature. The value *n* corresponds to the total number of studies for each group.

One bar chart, summarizing the most studied features in the ophthalmic and nonophthalmic disease groups, is presented in Figure 3. Hence, in the following sections, a detailed analysis on how these two biomarkers have been measured is provided. Moreover, a detailed explanation on how LCD and LCT measurements were carried out in each study is presented in Table 2. The normative values for the groups are also presented and discussed. In cases where more than one measurement was performed for the same feature (e.g., in different planes (superior, middle, or inferior), different scan directions (vertical and horizontal), or 2 eyes (left and right)), the pooled mean and SD were determined according to equations (1) and (2).

Only diseases for which at least 20 eyes were included in the studied group (cumulatively over all the evaluated papers) were considered for the average calculations (equations (1) and (2)).

### 3.1. LCT Measurements

**Figure 4:**
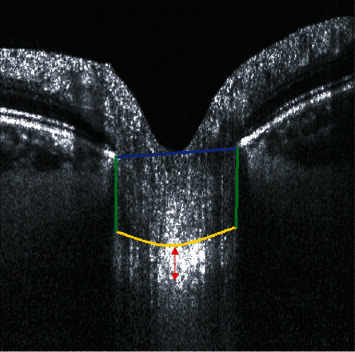
OCT B-scan showing the points used to compute the LCD. Line D in blue connects the two Bruch's membrane opening (BMO) edges. The two lines perpendicular to D in green are defined from the BMO edges to the anterior LC insertion points. The anterior LC surface is demarcated in yellow, enclosing the area S where the LC is measured. The red arrow measures the distance between the anterior and posterior margins of the LC, defined as the LCT.

LCT has been defined in the literature as the distance between the anterior and posterior borders of the highly reflective region visible below the optic disc cup in B-scan cross sections of the ONH (see the red arrow in Figure 4) [18]. However, a discrepancy between the LCT measurements, namely, between the locations used to calculate the LCT average, has been observed and reported in Table 2. For example, Lee et al. [74] considered three locations in each eye (midhorizontal, superior, and inferior midperipheral), with a separation of 100 *μ*m between the points. Bartolomé et al. [19] determined the points as close as possible to the vertical center of the ONH, which was identified as the point where the trunk of central retinal vessels extends from the ONH, as reported by Park et al. [12]. Other authors, such as Xiao et al. [20], considered LCT as the average of the central and paracentral points (150 *μ*m from the center point in the horizontal and vertical directions).

### 3.2. LCD Measurements

In the literature, LCD (also named as anterior lamina cribrosa depth in several studies) is defined as the perpendicular distance from the BMO plane to the maximum depth point of the anterior LC surface. All articles included in this review provide the measurements relative to Bruch's membrane opening (BMO). Only the measurements relative to BMO were considered for the calculations since all articles provide the measurement relative to this plane. Two studies, Rhodes et al. [21] and Luo et al. [22], also considered the scleral plane and the ASCO as the reference for depth measurements. These differences have been shown to lead to measurement bias, as reported by Luo et al. [22], who obtained 402 ± 91 *μ*m, 309 ± 88 *μ*m, and 332 ± 96 *μ*m for BMO, ASCO, and scleral reference planes, respectively. The number of selected points and B-scan planes to average the measure also influence the precision of the results. Other authors, such as Park et al. [23], obtained measurements as the average from 11 equidistant planes that divided the optic disc diameter into 12 equal parts vertically in each eye. A line was drawn from each of the two LC insertion points perpendicularly to the line connecting the two Bruch's membrane edges (see line D in Figure 4). The area surrounded by these two lines was measured (see area S in Figure 4). The mean LCD was approximated by dividing S by the length of D for each of the 11 horizontal OCT scans. Finally, Lee et al. [24] defined LCD as the mean of 3 values obtained from the 3 upper B-scans (1st to the 3rd scan), the 3 central B-scans (5th to the 7th scan), and at the 3 lower B-scans (from the 9th to the 11th scan) passing through the ONH. Commonly, temporal adjacent points were selected because the maximally depressed point was often close to the central vessel trunk, and its shadow obscured the LC [25].

### 3.3. Features' Applicability and Measurements

**Figure 5:**
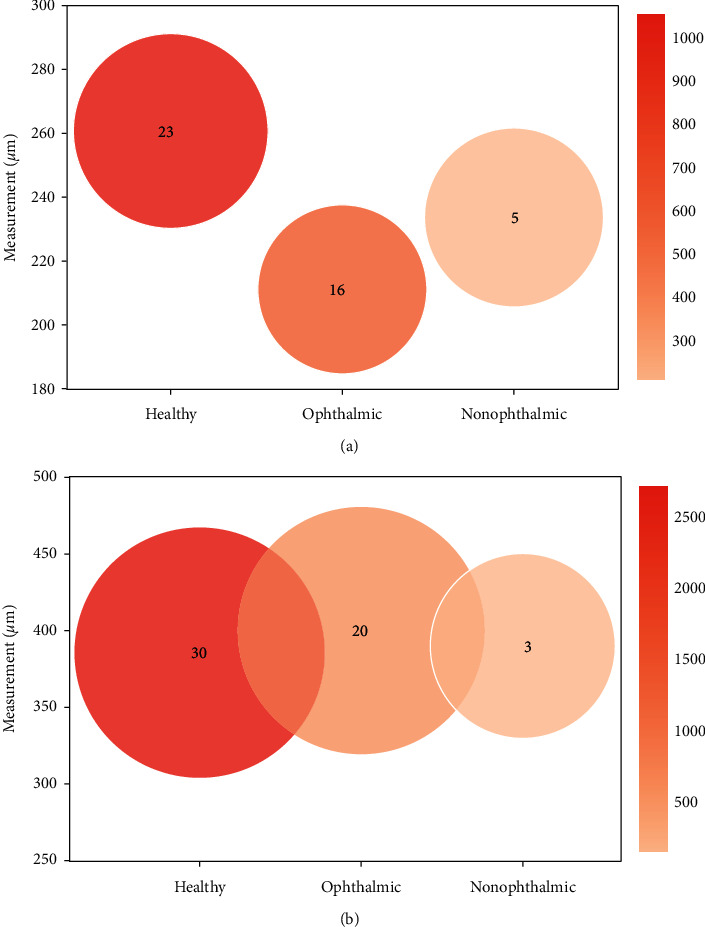
Comparison of lamina cribrosa (a) thickness (LCT) and (b) depth (LCD) between healthy controls, ophthalmic, and nonophthalmic diseases. The number in the circle represents the amount of studies used for calculating the averaged measurements for each group. The color scale shows the number of eyes comprised in the studied groups, and the radius of each circle denotes the standard deviation of the averaged values.

This section details the mean values for the two dominating LC structural features (LCT and LCD) in the three groups (healthy controls, ophthalmic, and nonophthalmic diseases). The values were calculated based on the articles presented in Table 2 for each group and disease, and the mean and SD values for each group are summarized in Figure 5.

#### 3.3.1. Healthy Group Measurements

Analysis of healthy subjects is very important to establish normative values for the healthy population, and hence facilitate the diagnosis and follow-up of the pathology. The studies that included only healthy subjects, as well as those comparing patients to a healthy control group, were selected, and the LCT and LCD average values were determined. The observed averages were 261 ± 39 *μ*m (range: 211–323 *μ*m) and 386 ± 91 *μ*m (range: 293–441 *μ*m) for the LCT and LCD, respectively. Figures 5(a) and 5(b) show a comparison between the three groups for both features. These parameters seem to be influenced by several factors, such as age and racial ancestry. Rhodes et al. [21] conducted a study in healthy eyes and concluded that the LC was significantly anteriorly displaced with increasing age in those with European ancestry in contrast to those with African ancestry.

#### 3.3.2. Ophthalmic Disease Group Measurements

**Figure 6:**
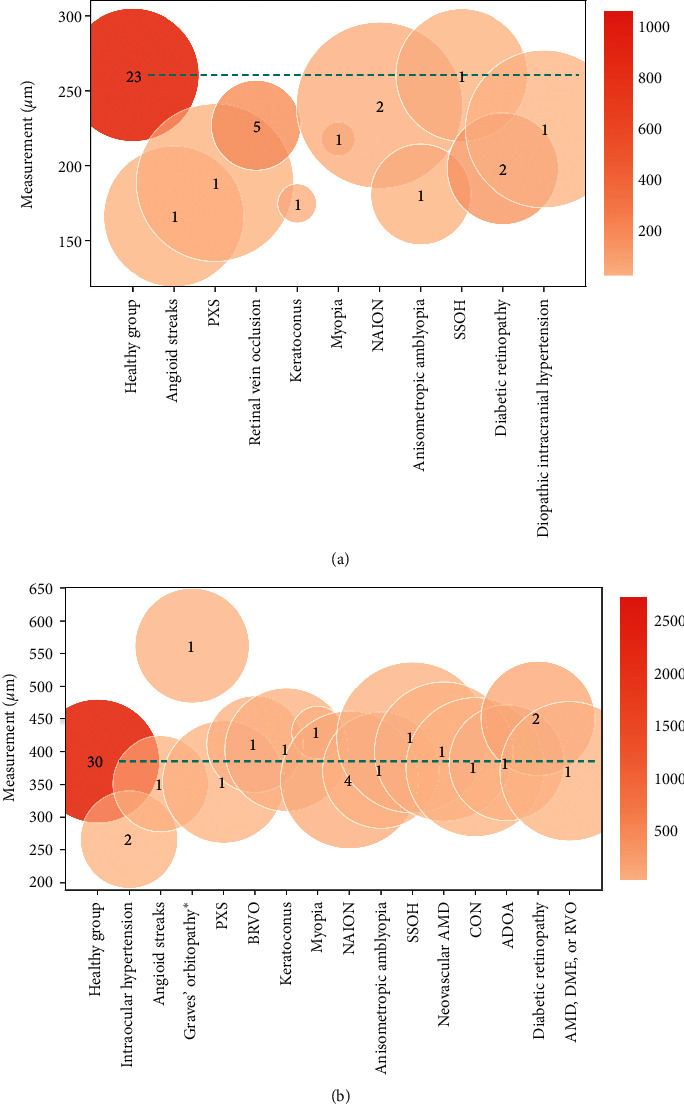
Comparison of lamina cribrosa (a) thickness (LCT) and (b) depth (LCD) for the ophthalmic disease group. The dashed green line represents the mean for the healthy population. Only pathologies that included more than 20 eyes among all articles are considered for the analysis.  ^*∗*^With proptosis and/or compressive optic neuropathy.

The ophthalmic disease group represented the largest group (*n* = 31) and included a large number of conditions, the most common being myopia, retinal vein occlusion (RVO), nonarteritic anterior ischaemic optic neuropathy (NAION), pseudoexfoliation syndrome (PXS), superior segmental optic nerve hypoplasia (SSOH), compressive optic neuropathy (CON), age-related macular degeneration (AMD), autosomal dominant optic atrophy (ADOA), and diabetic macular edema (DME). For ophthalmic patients, mean LCT and LCD were 211 ± 33 *μ*m and 403 ± 90 *μ*m, respectively (Figure 5). The graphics in Figure 6 presents the mean values for different ophthalmic diseases in comparison with the healthy population (horizontal dashed green line). Regarding the LCT, its mean was lower for every pathology in this group except for SSOH (Figure 6(a)). Overall, the studies that reported LCD in nonglaucomatous ophthalmic diseases showed a slightly higher mean LCD compared with healthy controls (Figure 5(b)). Nonetheless, this trend is not significant because the standard deviations of all the diseases cross the standard deviation of the healthy LCD (Figure 6(b)). For example, Rebolleda et al. [26] found a lower average LCD in the superior, middle, and inferior planes in eyes with NAION compared to healthy eyes (*p* < 0.001), which was also true between unaffected fellow eyes when compared to healthy eyes (*p* < 0.05). The maximum value for LCD was found in cases of Graves' orbitopathy with proptosis and/or compressive optic neuropathy. Seo et al. [27] conducted a study in these patients and reported 462.79 ± 95.96 *μ*m and 621.39 ± 78.39 *μ*m values, at baseline, for the muscle-dominant and fat-dominant group, respectively.

#### 3.3.3. Nonophthalmic Disease Group Measurements

**Figure 7:**
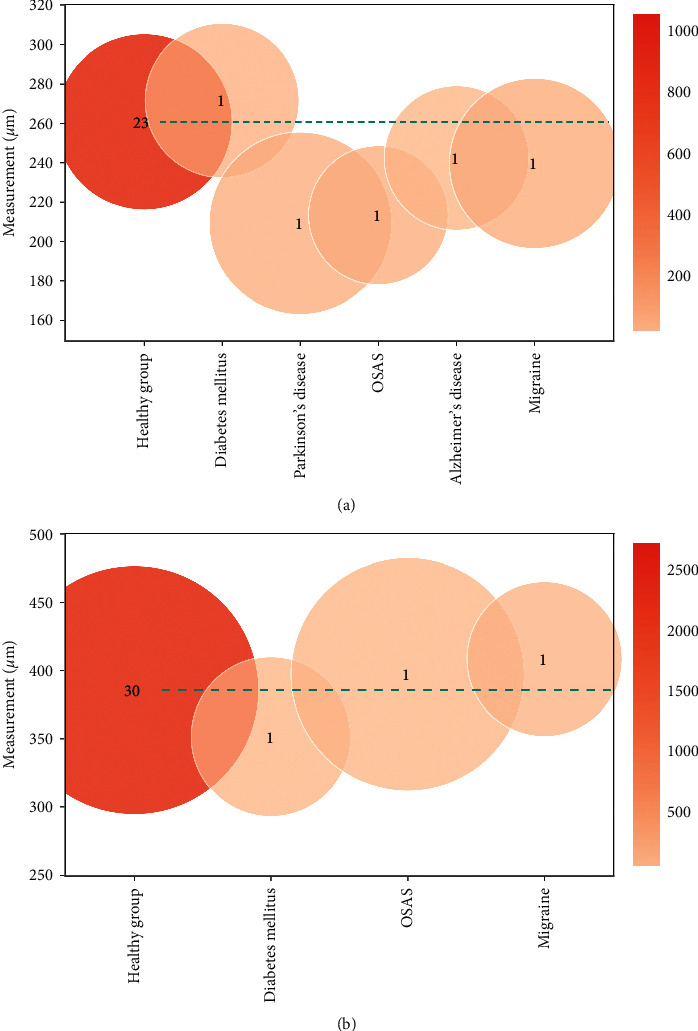
Comparison of lamina cribrosa (a) thickness (LCT) and (b) depth (LCD) for the nonophthalmic disease group. The green dashed line represents the mean for the healthy population. Only pathologies that included more than 20 eyes among all articles are considered for the analysis.

The number of studies in this group was smaller than in the other groups. The registered diseases were diabetes mellitus [28], Parkinson's disease (PD) [29], obstructive sleep apnea syndrome (OSAS) [30], Alzheimer's disease (AD) [31, 32], mild cognitive impairment (MCI) [31], and migraine [33]. The mean LCT and LCD were 234 ± 36 *μ*m and 390 ± 68 *μ*m, respectively, as shown in Figure 5. The graphics in Figure 7 presents LCT and LCD for each pathology in comparison to the healthy population (horizontal dashed green line).

LCT measurements seem to be lower relative to the healthy group, with the exception of diabetes mellitus. Akkaya et al. [28] described a significantly higher mean LCT in diabetic patients when compared to a healthy group, 271.61 ± 33.96 *μ*m vs. 248.50 ± 5.40 *μ*m, respectively (*p* < 0.001). Regarding LCD, diabetes mellitus showed significantly lower mean values in comparison to healthy controls in one study [28] (351 ± 59 *μ*m vs. 420 ± 90 *μ*m; *p*=0.003). The maximum mean LCD absolute value (deepest ONH cup) was described in patients with migraine (see Figure 7(b)). Sirakaya et al. [33] reported significantly higher mean LCD values for both migraine groups (412.15 ± 58.80 *μ*m with aura and 405.57 ± 55.39 *μ*m without aura) when compared to the healthy group (355.34 ± 65.53 *μ*m; *p*=0.001).

## 4. Discussion

The present study highlights which LC structural parameters have been analyzed in the literature with a focus on nonglaucomatous diseases. Overall, the most commonly studied parameters were LCD and LCT. The disease groups (ophthalmic and nonophthalmic) presented lower values for mean LCT, relative to the healthy population (Figure 5(a)). In parallel, mean LCD values were higher (deeper ONH cup) for these groups (Figure 5(b)). An exception in the nonophthalmic disease group was DM, which presented a shallower cup and thicker LC, when compared to healthy subjects. Akkaya et al. proposed that this evidence supports the “neuroprotective effect of DM on glaucomatous optic neuropathy and suggests that LCT and lamina cribrosa position mediate this protective effect.” [28]

This study shows that LC structural features are significantly different between healthy patients and some (nonglaucomatous) ocular and systemic pathologies. As such, there is a potential to add them as additional clinical features for clinical diagnosis. Nonetheless, being patient-specific features, LC features might hold an even better role for patient follow-up, signalling disease status' change. Unfortunately, we did not find any longitudinal studies focusing on this matter in this review. This fact highlights the need for longitudinal studies linking LC parameters and diseases, similarly to what is now common in glaucoma-related studies [75].

LC features are influenced by factors such as age, race, and also by the way measurements are carried out. However, these factors were not used as segmentation criteria in this study due to the lack of this information in several studies. For future works in this field, it is important to take into account these factors when analysing and comparing results between studies since they are a potential source of bias. Moreover, the current methods are heterogeneous (see Table 2), which may lead to imprecise comparisons between studies. For depth measurements, the consensus is to use the BMO plane as a reference, but the way the feature is measured is not consistent among research groups. One of the causes for this heterogeneity is the fact that the analysis of LC features still requires a considerable amount of manual input. This causes measurement bias due to the inherent difficulty of the manual delineation of the structure. This lack of automation increases the likelihood that each research group adopts their own reference points and methods. Besides, studies usually report averages of a limited number of B-scans without capturing the whole LC. The distance between these B-scan slices, as well as their number and position, may also be a source of discrepancies when comparing studies. Finally, some authors have pointed out the fact that the BMO reference place might be biased due to choroidal thickness changes and that perhaps the anterior sclera reference plane would be better suited for these calculations [76, 77]. Ideally, similar measurement methods should be adopted across all research groups. Currently, the measurement of the LC features is laborious and time consuming. As such, automation might hold the key to reduce bias in LC feature measurement. There is a need for easy-to-use software that can automatically measure LC features (possibly starting with LCT and LCD), ideally capturing all the information from OCT volumes, instead of selecting some of the B-scans. Providing such a tool with a fast and repeatable computation would contribute to making LC features a part of everyday clinical practice.

The main limitation of this review is the reduced number of studies, mainly in the nonophthalmic disease group, which precludes definite conclusions. Moreover, due to the lack of individual study data, it was not possible to perform statistical comparisons between groups and pathologies. As such, our results point towards differences that need to be better clarified. Nonetheless, LC features' ability to discriminate between these groups is supported by results presented by several individual studies, as reported in Table 2. Lastly, it is noteworthy to mention that the statistical analysis performed on groups (ophthalmic and nonophthalmic diseases) may be biased by different pathologies comprised in each group.

## 5. Conclusion

There is a growing interest in LC features outside the glaucoma field. The results of this meta-analysis show several promising features (mainly, LCT and LCD) that may be relevant for clinical practice. Nevertheless, further studies are needed to validate these findings, and longitudinal data are needed to clarify the potential for use in patient follow-up. Moreover, efforts should be employed to develop automated tools that can capture LC features from OCT data in a standardized manner, thus allowing more accurate comparisons between studies. These efforts should enable to further explore the potential of LC parameters for use in daily clinical practice.

## Figures and Tables

**Table 1:** Characteristics of the reviewed studies.

Authors	Group type	Ophthalmic disease	Parameter	No. of patients/eyes	Age (years) and *p* value	Technique	Device	Light WL (nm)	Image quality cutoff	Field of view
Xiao et al. [20]	Healthy subjects	—	cLCT cALCSD cPTT	Total: 96/96 Control: — Exp.: 96/96	YG: 26.72 ± 6.26 MG: 49.44 ± 5.64 OG: 67.19 ± 6.04 Mean: 50.34 ± 18.20 *p*=0.64	EDI with SD-OCT	Spectralis	870	Poor image quality affecting the recognition of the boundary of the LC.	Radial scanning protocol comprising 6 angularly equidistant linear scans centering at the center of ONH (scan angle: 20°).

Sousa et al. [34]	Healthy subjects	—	ALCD	Total: 59/59 Control: — Exp.: 59/59	61.7 ± 15.1	EDI with SD-OCT	Spectralis	870	<15.	A 20° 5.8 × 5.8 mm square covering the optic disc.

Lee et al. [35]	Healthy subjects	—	LCT	Total: 100/189 Control: — Exp.: 100/189	48.6 ± 13.9	EDI with SD-OCT	Spectralis	870	Eyes were excluded when a good-quality image (i.e., quality score >15) could not be obtained for more than five sections. Images did not allow clear delineation of both anterior and posterior borders of the central LC.	Scan line distance is determined automatically by the machine at the center of the ONH.

Bartolomé et al. [19]	Healthy subjects	—	LCD LCT	Total: 81/81 Control: — Exp.: 81/81	28.05 ± 8.4	EDI with SD-OCT	Spectralis	840	Very small optic disc, media opacity, prominent vascular shadow, or significant artefacts.	A 15° × 10° vertical rectangle centered on the optic disc.

Leal et al. [36]	Healthy subjects	—	ALCD	Total: 61/120 Control: — Exp.: 61/120	62.1 ± 15.01	EDI with SD-OCT	Spectralis	870	<15.	A 20° 5.8 × 5.8 mm square covering the optic disc; 2 perpendicular cross-scans (vertical and horizontal) intersected in the optic disc center.

Rhodes et al. [21]	Healthy subjects of African descent (AD) and European descent (ED)	—	LCD	Total: 84/166 Control: — Exp.: 56 AD young 8 AD old 54 ED young 48 AD old *p* < 0.001 between the AD and ED group	58.4 ± 15.5	EDI with SD-OCT	Spectralis	870	<20. Poor centration of the ONH.	20° radial scans centered on the ONH.

Wang et al. [37]	Healthy subjects	—	LC strains (deformations): (i) LC displacements (ii) LC strains	Total: 16/16 Control: — Exp.: 11 PPA 5 non-PPA	25 ± 3	EDI with SD-OCT	Spectralis	870	Poor visibility of the LC.	Rectangular region of 15° × 10° centered on the ONH.

Kim et al. [38]	Healthy subjects	—	LC displacement ALCD	Total: 48/48 Control: — Exp.: 48/48	25.6 ± 5.4	EDI with SD-OCT	Carl Zeiss Meditec	NA	<7. Unclear visibility of more than one-quarter of the anterior LC surface of the neural canal opening diameter.	200 × 200 optic disc cube scan; 5-HD line scans (6 mm length) centered to optic disc; and 1-HD line scan (9 mm length) aligned to the axis connecting the fovea and the center of the optic disc.

Poli et al. [39]	Healthy subjects	—	ALCSD	Total: 8/16 Control: — Exp.: 8/16	52	SS-OCT	Topcon	1050	Good-quality image (i.e., mean success score rate >96/128) allowing clear delineation of the anterior borders of the LC and the posterior border of the choroid.	Horizontal 6-mm line scan centered at the optic disc.

El-Agamy et al. [40]	Healthy subjects	—	ALCSD	Total: 191/191 Control: — Exp.: 191/191	20.76 ± 1.627	SD-OCT	Topcon	840	≤56.	6 × 6 mm square covering the optic disc.

Park et al. [23]	Healthy subjects	—	LCD	Total: 30/30 Control: — Exp.: 30/30	40 ± 18	EDI with SD-OCT	Spectralis	870	Poor quality because of media opacity or poor patient cooperation, causing diffusely unclear images or significant artifacts.	15° × 10° rectangle for horizontal scans (10° × 15° rectangle for vertical scans) centered on the optic disc.

Lee et al. [24]	Healthy subjects	—	ALCD LCT	Total: 26/26 Control: — Exp.: 26/26	63.4 ± 8.0	EDI with SD-OCT	Spectralis	870	Poor B-scan quality that did not allow the delineation of the borders of the LC.	Vertical and horizontal B-scan images covering the optic disc, separated by 30–34 *μ*m (the scan-line distance being determined automatically by the instrument).

Bedggood et al. [41]	Healthy subjects	—	Axial shifts of the anterior LC	Total: 21/21 Control: — Exp.: 21/21	33.3 ± 6.8	EDI with SD-OCT	Spectralis	870	<25.	Cubes of extent 10° × 15° (horizontal × vertical), with vertical B-scans 0.21° apart (∼60 *μ*m) centered on the optic nerve head and oriented vertically.

Lee et al. [42]	Healthy subjects	—	LCCI	Total: 125/250 Control: — Exp.: 125/250	49.02 ± 14.13	EDI with SD-OCT	Spectralis	870	<15.	A 10° × 15° rectangle covering of the optic disc.

Seo et al. [43]	Healthy subjects	—	ALCD	Total: 150/300 Control: — Exp.: 150/300	48.31 ± 14.31	EDI with SD-OCT	Spectralis	870	Quality score >15 could not be obtained at more than five sections.	A 10° × 15° rectangle covering the optic disc.

Fazio et al. [44]	Healthy population with different descendents	—	ALCS displacement and depth	Total: 21/42 Control: — Exp.: 12/24 AD 9/18 ED	55.8 ED 45.2 AD *p*=0.065	EDI with SD-OCT	Spectralis	870	<20. Poor centration of the optic nerve head.	Radial scans centered on the center of the optic nerve.

Tun et al. [45]	Healthy subjects	—	LCD	Total: 619/619 Control: — Exp.: 619/619	60.23 ± 7.36	EDI with SD-OCT	Spectralis	870	OCT images with a poor scleral visibility.	15° × 10° rectangle centered on the ONH.

Thakku et al. [46]	Healthy subjects	—	LC morphology: (i) LC depth (ii) LC curvature	Total: 162/162 Control: — Exp.: 162	58 ± 7	EDI with SD-OCT	Spectralis	870	Poorly visible LC (LC covering less than 70% of the BMO area from en face visualization).	15° × 15° rectangular region centered on the ONH.

Luo et al. [22]	Healthy subjects	—	cLCD	Total: 360/360 Control: — Exp.: 357 visible sclera 344 visible lamina 339 visible sclera and lamina	50.6 ± 17.5	EDI with SD-OCT	Spectralis	870	<20.	15° B-scans centered on BMO.

Akkaya et al. [28]	Not ophthalmic Diabetes mellitus	—	ALCD LCT	Total: 70/70 Control: 32/32 Exp.: 38/38	59.1 ± 7.4 diabetes group 58.6 ± 7.7 control group *p*=0.696	EDI with SD-OCT	Spectralis	870	<20. Unclear image of the fundus or the border of the lamina cribrosa.	15° × 10° rectangle centered on the optic disc.

Eraslan et al. [29]	Not ophthalmic Parkinson's disease (PD)	—	LCT	Total: 47/94 Control: 25/50 Exp.: 22/44	60.56 ± 9.9 control group 60.45 ± 9.1 PD patients *p*=0.976	SD-OCT	Optovue	840	<50.	6 × 6 mm. The scans passing through the center of the central retinal blood vessels were centered at the optic disc with nasal fixation.

Küçük et al. [30]	Not ophthalmic Obstructive sleep apnea syndrome (OSAS)	—	LCT LCD	Total: 88/88 Control: 43 Exp.: 45 13 mild OSAS 17 moderate OSAS 15 severe OSAS	50.30 ± 4.2 control group 50.09 ± 9.7 experimental group *p*=0.931	EDI with SD-OCT	Spectralis	870	NA.	A 15° × 10° rectangular image centered on the optic disc.

López-de Eguileta et al. [31]	Not ophthalmic Alzheimer's disease (AD) Mild cognitive impairment (MCI)	—	PTT ALCSD	Total: 66/126 Control: 63 Exp.: 12 AD 51 MCI	73.28 ± 6.0 control group 73.5 ± 6.0 experimental group *p*=0.998	SD-OCT	Spectralis	870	The quality of the scans is indicated on a color scale at the bottom of the scanned images. Only scans in the green range were considered of sufficiently good quality for inclusion.	A 15° area scan centered at the ONH.

Lee et al. [32]	Not ophthalmic Alzheimer's disease (AD)	—	LCT	Total: 44/44 Control: 26/26 Exp.: 18/18	63.4 ± 8.0 control group 69.7 ± 7.6 experimental group *p*=0.012	EDI with SD-OCT	Spectralis	870	B-scan quality that did not allow delineation of the LC borders.	Horizontal and vertical B-scan images covering the optic disc, 30–34 *μ*m apart.

Sirakaya et al. [33]	Not ophthalmic Migraine	—	LCT LCD	Total: 97/97 Control: 35 (group III) Exp.: 27 migraine with aura (group I) 35 migraine patients without aura (group II)	33.0 ± 4.7 group I 33.9 ± 5.4 group II 32.4 ± 4.8 group III *p*=0.460	EDI with SD-OCT	Spectralis	870	<20.	A 15° × 10° rectangular image centered on the ONH.

Pasaoglu et al. [47]	Ophthalmic	Diopathic intracranial hypertension (IID)	LCT ALCSD	Total: 18/36 Control: 10/20 Exp.: 8/16	NA control group 41.1 ± 7.1 experimental group	DRI with SS-OCT	Topcon	1050	NA.	11-horizontal line raster scan protocol.

Villarruel et al. [25]	Ophthalmic	Intracranial hypertension (IIH) Primary open-angle glaucoma (high-tension glaucoma (HTG) and normal-tension glaucoma (NTG))	LCD	Total: 61/88 Control: 37 Exp.: 11 IIH 20 HTG 20 NTG	24.3 ± 4.8 IIH patients 69.8 ± 10.2 glaucoma patients	EDI with SD-OCT	Spectralis	870	<15.	20° × 10° rectangle scanning covering the ONH.

García-Montesinos et al. [48]	Ophthalmic	Papilledema	LCD PTT	Total: 8/12 Control: — Exp.:6 TLCD <−9.2 mmHg (group 1) 6 TLCD >−9.2 mmHg (group 2)	39 ± 19.9	EDI with SD-OCT	Spectralis	870	Inaccurate images owing to segmentation algorithm errors (failed to detect the edges of the BMO).	Vertical scan closest to the ONH center and where the visibility of the anterior LC surface was more complete.

Demir et al. [49]	Ophthalmic	Angioid streaks (AS)	ALCD LCT	Total: 74/74 Control: 42/42 Exp.: 32/32	53.8 ± 10.2 control group 51.7 ± 8.0 AS patients *p*=0.34	EDI with SD-OCT	Spectralis	870	NA.	Single line scan centered on the optic disc.

Seo et al. [27]	Ophthalmic	Graves' orbitopathy (GO)	LCD	Total: 42/69 Control: — Exp.: 40 (excluded 19) 15 muscle-dominant 25 fat-dominant	45.2 ± 4.01 muscle-dominant 31.8 ± 1.9 fat-dominant *p*=0.002	EDI with SD-OCT	Spectralis	870	B-scan images were not well visualized to discriminate the anterior surface of the LC.	15° × 10° covering the optic disc.

Moghimi et al. [50]	Ophthalmic	Pseudoexfoliation syndrome (PXS)	LCT ALD PLD	Total: 61/61 Control: 29/29 Exp.: 32/32	64.86 ± 7.04 control group 67.94 ± 7.30 PXS group *p*=0.10	EDI with SD-OCT	Spectralis	870	<20 inadequate quality as determined by unclear fundus images, interruption of the RNFL, or unclear border of the LC or posterior choroid.	A 15° × 10° rectangle centered on the optic disc.

Soares et al. [51]	Ophthalmic	Spontaneous intracranial hypotension	ALCD	Total: 10/20 Control: 10/10 Exp.: 10/10	36.8 ± 4.6 control group 38 ± 4.18 experimental group	EDI with SD-OCT	Spectralis	870	NA.	B-scan images obtained by dividing the optic disc into 48 equal diagonal slices.

Karaca Adıyeke et al. [52]	Ophthalmic	Central retinal vein occlusion (CRVO)	LCT	Total: 67/67 Control: 35/35 Exp.: 32/32	57.9 ± 13.8 control group 62.2 ± 11.6 experimental group	EDI with SD-OCT	Spectralis	870	Vertical scans without clearly visible borders.	Vertical scans with clearly visible borders centered on ONH.

Son et al. [53]	Ophthalmic	Branch retinal vein occlusion (BRVO)	LCT PTT	Total: 85/85 Control: 35/35 Exp.: 50/50	59.4 ± 12.9 control group 60.9 ± 12.2 experimental group *p*=0.615	EDI with SD-OCT	Spectralis	870	The lamina cribrosa margin was not defined.	10° × 15° covering the optic disc.

Sırakaya and Bekir [54]	Ophthalmic	Unilateral branch retinal vein occlusion (BRVO)	LCT LCD	Total: 73/108 Control: 38 Exp.: 35	65.15 ± 9.85 control group 65.48 ± 8.91 experimental group *p*=0.882	EDI with SD-OCT	Spectralis	870	<20.	A 15° × 10° rectangular image centered on the optic disc.

Altunel et al. [55]	Ophthalmic	Central retinal vein occlusion	LCT	Total: 80/80 Control: 42/42 Exp.: 38/38	64.5 ± 10.3 control group 65.2 ± 11.0 experimental group *p*=0.781	EDI with SD-OCT	Spectralis	870	Scans with invisible LC borders.	A 10° × 15° rectangle covering the optic disc.

Lim et al. [56]	Ophthalmic	Unilateral branch retinal vein occlusion (BRVO)	LCT	Total: 77/77 Control: 31/31 Exp.: 46/46	61.8 ± 11.6 control group 60.5 ± 11.1 experimental group *p*=0.635	EDI with SD-OCT	Spectralis	870	<15.	A 15° × 10° rectangular image centered on the ONH.

Akkaya and Küçük [57]	Ophthalmic	Keratoconus	LCT LCD	Total: 101/101 Control: 56/56 Exp.: 45/45	22.5 ± 7.4 control group 24.5 ± 7.2 experimental group *p*=0.17	EDI with SD-OCT	Spectralis	870	<20.	A 15° × 10° rectangular image centered on the optic disc.

Lee et al. [58]	Ophthalmic	Myopia	LCT LCD PTT	Total: 40/40 Control: — Exp.: 40/40	28 ± 9	EDI with SD-OCT	Spectralis	870	Images with unclear LC margin, severe shadowing due to overlying vessels, or poor image quality due to cataracts.	A 15° × 10° rectangular image centered on the optic disc.

Jnawali et al. [59]	Ophthalmic	Myopia	ALCSD	Total: 52/52 Control: 29/29 Exp.: 23/23	10.16 ± 2.48 control group 12.43 ± 2.31 experimental group *p*=0.001	EDI with SD-OCT	Spectralis	880	<30.	A 12° peripapillary circular scan centered at the optic nerve head.

Ohno-Matsui et al. [60]	Ophthalmic	Myopia	Distance between the inner surface of the LC and the subarachnoid space (SAS)	Total: 108/165 Control: 32 Exp.: 133	Patients with pathologic myopia: 53.2 ± 11.9 subarachnoid space of the optic nerve visible by OCT 59.7 ± 9.3 subarachnoid space of the optic nerve not visible by OCT	SS-OCT	Topcon	1050	Poor image quality because of dense cataract, poor fixation because of macular chorioretinal atrophy, myopic macular holes, or severe visual field defects.	3 × 3 mm and 6 × 6 mm scans centered on the optic disc.

Miki et al. [61]	Ophthalmic	Myopia glaucoma	LC defects	Total: 108/159 Control: 35 Exp.: 67 high myopia with glaucoma (MG group) 22 glaucoma without high myopia (G group) 35 high myopia without glaucoma (M group)	57.4 ± 16.6 control group 52.4 ± 12.4 MG group 62.3 ± 10.4 G group 55.0 ± 13.5 M group *p*=0.1076	SS-OCT	Topcon	1600	Poor quality images such as poor contrast images due to media opacity or poorly fixated images. Eyes with poor visibility of the LC, defined as less than 80% visibility of the anterior laminar surface within the ONH area.	A 6 × 6 mm cube centered on the ONH.

Han et al. [18]	Ophthalmic	Myopia with and without open-angle glaucoma	LC defects	Total: 282/282 Control: 58 Exp.: 90 myopic eyes without OAG 134 myopic eyes with OAG	51.3 ± 10.7 control group 46.1 ± 11.0 myopic eyes without OAG 50.9 ± 11.0 myopic eyes with OAG *p*=0.35	EDI with SD-OCT	Spectralis	870	Scan with <70% of the anterior LC visible due to prelaminar tissue, RPE, or overlying vessels in ≥3 of the 48 radial line scans.	Radial line B-scans (each at an angle of 3.75°) centered on the optic disc.

Rebolleda et al. [62]	Ophthalmic	Nonarteritic anterior ischaemic optic neuropathy (NAION)	ALCSD LCT PTT	Total: 34/34 Control: 17/17 Exp.: 17/17	71.9 ± 10.7 NAION patients	EDI with SD-OCT	Spectralis	870	Inaccurate images due to errors in the segmentation algorithm (failed to detect the edges of the BMO). Scan without retinal vasculature and where borders were more clearly visible was evaluated.	Vertical scan that was closest to the ONH center and where the visibility of the anterior LC surface was complete (without including main vessels).

Fard et al. [63]	Ophthalmic	Nonarteritic anterior ischaemic optic neuropathy (NAION) Primary open-angle glaucoma (POAG)	ALCS LCT	Total: 91/121 Control: 29 Exp.: 32 POAG 30 NAION patients and their 30 fellow eyes	63.4 ± 7.9 control group 65.6 ± 13.1 POAG 58.4 ± 10.5 NAION *p*=0.01	EDI with SD-OCT	Spectralis	870	Images with poor centration, segmentation errors, or poor quality (<15 dB).	A 15° × 15° square covering the optic disc.

Lee et al. [64]	Ophthalmic	Normal-tension glaucoma (NTG) Nonarteritic anterior ischemic optic neuropathy (NAION)	LCD PTT	Total: 105/105 Control: 42 Exp.: 21 NAION 42 NTG	60.0 ± 9.7 control group 60.4 ± 9.8 NTG 61.6 ± 10.6 NAION *p*=0.686	EDI with SD-OCT	Spectralis	870	<15 scans that did not allow clear delineation of the anterior border of the LC.	A 10° × 15° rectangle covering the optic disc.

Rebolleda et al. [26]	Ophthalmic	Nonarteritic anterior ischemic optic (NAION) Primary open-angle glaucoma (POAG)	ALCD PTT	Total: 68/88 Control: 23 Exp.: total: 65 23 NAION 17 fellow unaffected eyes 25 POAG	68.6 ± 10.7 control group 68.6 ± 10.3 NAION 72.3 ± 9.8 POAG *p*=0.53	EDI with SD-OCT	Spectralis	870	Segmentation errors, poor centration, or poor quality.	A 15° × 10° vertical rectangle centered on the optic disc.

Akkaya et al. [28]	Ophthalmic	Anisometropic Amblyopia	LCT LCD	Total: 95/127 Control: 32 Exp.: 32 hyperopic anisometropic amblyopia and 32 fellow eyes 31 hyperopic nonamblyopic	11.2 ± 2.0 control group 12.0 ± 1.8 amblyopic and fellow eyes 10.7 ± 2.2 hyperopic nonamblyopic *p* (amblyopia vs. fellow) = 1.0 *p* (amblyopia vs. control) = 0.58 *p* (amblyopia vs. hyperopic nonamblyopia) = 0.15	EDI with SD-OCT	Spectralis	870	<20 unclear fundus images and unclear LCT border.	A 15° × 10° rectangular image centered on the optic disc.

Shinohara et al. [65]	Ophthalmic	Tilted disc syndrome (TDS)	Sloping of the lamina cribrosa	Total: 44/54 Control: 16 Exp.: 38	74.9 ± 9.3 control group 66.1 ± 11.2 experimental group	SS-OCT	Topcon	1050	NA.	Scan length: 12 mm centered on the optic disc.

Lee et al. [64]	Ophthalmic	Superior segmental optic nerve hypoplasia (SSOH) Primary open-angle glaucoma (POAG)	LCT LCD	Total: 126/126 Control: 54 Exp.: 35 SSOH 37 POAG	46.1 ± 15.2 control group 29.1 ± 12.5 SSOH 54.7 ± 12.1 POAG *p* < 0.001	EDI with SD-OCT	Spectralis	870	<15 images did not allow clear delineation of both anterior and posterior borders of the central portion of the LC.	Horizontal B-scan section images covering the optic disc, 30 to 34 *μ*m apart (the scan-line distance being determined automatically by the instrument).

Rebolleda et al. [66]	Ophthalmic	Neovascular age-related macular degeneration	LCD PTT	Total: 50/50 Control: 20/20 Exp.: 30/30	74.5 ± 7.7 control group 77.4 ± 6.8 experimental group *p*=0.432	EDI with SD-OCT	Spectralis	870	Only the highest-quality image and most centered vertical scan without retinal vasculature and where borders were more clearly visible were evaluated.	Vertical scan that was closest to the ONH center and where the visibility of the anterior LC surface was complete (excluding the main vessels).

Hata et al. [67]	Ophthalmic	Compressive optic neuropathy (CON) Glaucomatous optic neuropathy (GON)	LCD PTT	Total: 102/102 Control: 34 Exp.: 34 CON 34 glaucoma	59.4 ± 14.6 control group 59.2 ± 13.2 CON 59.5 ± 13.5 glaucoma *p*=0.94 (between CON and glaucoma) *p*=0.95 (between CON and normal subjects)	EDI with SD-OCT	Spectralis	870	NA.	Radial scanning pattern centered on the optic disc (24 high-resolution 15° radial scans).

Kim et al. [7]	Ophthalmic	Normal tension glaucoma (NTG) Autosomal dominant optic atrophy (ADOA)	LCCI LCD	Total: 120/120 Control: 48 Exp.: 24 ADOA 48 NTG	63.38 ± 14.45 control group 63.25 ± 15.66 ADOA 63.42 ± 12.74 NTG *p*=0.894	EDI with SD-OCT	Spectralis	870	<15.	A 10° × 15° rectangle covering the optic disc.

Yang et al. [68]	Ophthalmic	Diabetic retinopathy with and without panretinal photocoagulation	ALCSD LCT PTT	Total: 206/206 Control: 33 (group I) Exp.: 30 without diabetic retinopathy (group II), 66 nonproliferative diabetic retinopathy (group III), 45 panretinal photocoagulation (group IV), and 32 normal tension glaucoma (group V)	53.5 ± 8.8 group I 55.7 ± 12.3 group II 56.6 ± 10.4 group III 56.2 ± 9.9 group IV 53.8 ± 13.0 group V *p*=0.595	SS-OCT	Topcon	1500	Unable to visualize the lamina cribrosa and/or peripapillary tissue clearly.	Scans with a scan length of 6 × 6 mm.

Yokota et al. [69]	Ophthalmic	Neovascular glaucoma (NVG) Diabetic retinopathy	ALCT LCT	Total: 46/46 Control: — Exp.: 20 PDR with non-NVG 26 PDR with NVG	66.2 ± 2.4 PDR with the non-NVG group 61.4 ± 2.1 PDR with the NVG group *p*=0.151	EDI with SD-OCT	Spectralis	870	Eyes with other ocular diseases that might decrease the image quality of OCT were excluded (e.g., vitreous hemorrhage).	Horizontal B-scans at an interval of 50 *μ*m.

Gómez-Mariscal et al. [70]	Ophthalmic	Age-related macular degeneration (AMD) Diabetic macular edema (DME) or retinal venous occlusion (RVO)	LCD PTT	Total: 29/53 Control: 24 Exp.: 29	76.9 ± 6.6 control group 76.8 ± 6.9 experimental group *p*=0.943	EDI with SD-OCT	Spectralis	870	Presence of media opacities which prevented a good image quality.	Vertical scan selected close to the center of the papilla, and three vertical measurements were performed to obtain a representative value of the relative position and displacement of ONH structures.

LC = lamina cribrosa, LCT = lamina cribrosa thickness, cLCT = central lamina cribrosa thickness, LCD = lamina cribrosa depth, cLCSD = central anterior lamina cribrosa surface depth, PTT =  prelaminar tissue thickness, cPTT = central prelaminar tissue thickness, Exp = experimental, YG = young group, MG = middle group, OG = old group, EDI = enhanced depth imaging, SD = spectral domain, SS = swept source, ALCSD = anterior lamina cribrosa surface depth, ONH = optic nerve head, PPA = peripapillary atrophy, LCCI = lamina cribrosa curvature index, PLD = posterior laminar depth, and NA = not available, that is, not mentioned in the article.

**Table 2:** Approach used for the measurement and reported values for the lamina cribrosa thickness and depth.

Authors	Measurement	Results
Xiao et al. [20]	(i) cCLT was defined as “the distance between the anterior lamina cribrosa surface (ALCactS) and posterior lamina cribrosa surface (PLCS). cLCT was calculated from the average value of the LCT in the ONH center point and paracentral points (150 *μ*m from the center point in the horizontal and vertical directions).” (ii) cALCSD was defined as “the distance between the ALCS and the reference plane (the connection of the terminal of Bruch's membrane was defined as the reference plane). The cALCSD was calculated from the average value of ALCSD in the ONH center point and paracentral points (150 *μ*m from the center point in the horizontal and vertical directions).”	(i) cLCT (*μ*m): 235.18 ± 41.27; *p* < 0.001 between the 3 groups (YG/MG/OG) (ii) cALCSD (*μ*m): 358.02 ± 93.80; *p*=0.11 between the 3 groups (YG/MG/OG)

Sousa et al. [34]	ALCD was defined as “the prependicular distance from the BMO plane to the maxium depth point of the anterior LC border. A mean of the 2 measurements was used.”	(i) Vertical scan (*μ*m): 456.2 ± 84.3 (right eye)/444.5 ± 92.2 (left eye); *p*=0.19 (ii) Horizontal scan (*μ*m): 436.7 ± 81.6 (right eye) 427.6 ± 82.7 (left eye); *p*=0.13 (iii) Pool mean + SD (*μ*m): 441.5 ± 82.5

Lee et al. [35]	“LCT was measured as the distance between the levels of the anterior and posterior borders in the B-scan images. LCT was measured at the midpoint between the opening of Bruch's membrane and two additional points that were 150 *μ*m from either side of the midpoint.”	273.19 ± 34.74 *μ*m

Bartolomé et al. [19]	(i) “LCD was measured in 11 horizontal B-scans that were spaced equally along the vertical diameter of the optic disc. The line connecting both Bruch's membrane opening (BMO) edges was used as a reference plane for all depth measurements. A line perpendicular to this reference line was drawn from each BMO edge to the anterior surface of the lamina cribrosa. When one of these two perpendicular lines did not meet the anterior laminar surface because of disc tilting and associated lateral lamina displacement, a line was drawn from the anterior lamina cribrosa insertion point perpendicularly to the line connecting the two BMO edges. The area defined by these two perpendicular lines, the line connecting the BMO edges and the anterior laminar surface, was measured area *S*. Mean LCD depth in each of the 11 horizontal EDI-OCT scans was defined by area *S* divided by length *D*.” (ii) LCT was defined as “the distance between the anterior and posterior borders of the highly reflective region at the vertical center of the ONH. LCT was determined as close as possible to the vertical center of the ONH, which was identified as the site where the trunk of central retinal vessels extends from the ONH.”	(i) LCD (*μ*m): 329.15 ± 60.85 (male: 351.03 ± 63.83/female: 319.01 ± 57.39; *p*=0.057) (ii) LCT (*μ*m): 323.25 ± 56.02 (male: 344.75 ± 48.73/female: 313.07 ± 71.14; *p*=0.094)

Leal et al. [36]	“ALCD was defined as the maximum perpendicular distance between the line connecting both ends of Bruch's membrane and the maximum depth point of the anterior border of the LC. The anterior border of the LC was defined by a highly reflective structure below the optic cup.”	(i) Right eye vertical (REV) scan (*μ*m): 456.16 ± 84.32 (ii) Left eye vertical (LEV) scan (*μ*m): 444.53 ± 92.19 (iii) Right eye horizontal (REH) scan (*μ*m): 436.66 ± 81.57 (iv) Left eye horizontal (LEH) scan (*μ*m): 427.56 ± 82.71 (v) Pool mean + SD (*μ*m): 441.23 ± 85.20

Rhodes et al. [21]	“LCD measures the distance of the LC from a reference plane, either a BMO plane (LDBMO) or a scleral plane (LDAS). The definition and computation of mean LD require the definition of a reference structure against which to measure depth, a surface reconstruction and sampling for mean depth, and the use of a suitable coordinate frame for the manually delineated point clouds. The Bruch structure (BMO plane, BMO ellipse, and laminar half-space) is computed, and the LC moved to a Bruch frame. The LC sections are uniformly resampled; an optimal mesh is built from these LC sections; and mean LDBMO is computed as a weighted average of the mesh centroid depths. The scleral structure is built using an interior disk-like region (the region of the AS between 1700 and 1800 *μ*m from the axis of the BMO cylinder, the right elliptic cylinder defined by the BMO ellipse, orthogonal to the BMO plane). Like the BMO, this region is almost planar. The scleral representative of an AS half-section is the mean of the samples that lie between 1700 and 1800 *μ*m from the axis of the BMO cylinder (using Euclidean, not geodesic distance). The mean is used (as opposed to the point at 1750 *μ*m, e.g.) as a smoothing operation. The scleral plane (ellipse) is the best-fitting plane (ellipse) of the point cloud of scleral representatives, analogous to the BMO plane/ellipse. Replacing the BMO plane by the scleral plane, we have LD based on a scleral reference plane (distance of LC from the scleral plane, LDAS). These measurements are again simplified by using a special frame, where depth becomes the *z*-coordinate.”	(i) LD BMO (*μ*m): 413.88 ± 75.06 (AD young) 365.38 ± 64.48 (AD old) 382.43 ± 93.29 (ED young) 337.73 ± 82.41 (ED old) Pool mean + SD: 379.29 ± 82.70 (ii) LD AS (*μ*m): 333.61 ± 83.84 (AD young) 353.75 ± 60.32 (AD old) 316.39 ± 87.60 (ED young) 309.20 ± 78.69 (ED old)

Kim et al. [38]	Anterior LC depth (LCD) was defined as “the maximal vertical distance between the reference plane connecting Bruch's membrane openings (BMO) and the anterior LC surface.”	463.4 ± 118.8 *μ*m

El-Agamy et al. [40]	”ALCSD was measured at all planes, defined as the distance from the line connecting the two Bruch's membrane opening (BMO) edges (reference line) to the anterior LC surface. It was measured in the direction perpendicular to the reference plane at three points: the maximum depth point and two additional points (100 and 200 *μ*m apart from the maximum depth point to the temporal direction). Only the temporally adjacent points were selected because the LC at the maximally depressed point was masked by the shadow of the central vessel trunk. The average of three measurements was taken as the ALCSD of each plane. The average of the ALCSDs from all planes was defined as the mean of ALCSD of the eye.”	(i) Mean of ALCSD (*μ*m): 371.88 ± 114.62 (ii) Superior plane (*μ*m): 368.08 ± 116.04 (iii) Middle plane (*μ*m): 379.86 ± 117.12 (iv) Inferior plane (*μ*m): 366.59 ± 120.98 Significant difference between superior and middle planes (*p*=0.004) and middle and inferior planes (*p*=0.013) but no significant difference between superior and inferior planes (*p*=0.820)

Lee et al. [24]	(i) “LCD was determined by measuring the distance from the Bruch's membrane (BM) opening plane to the level of the anterior LC surface in 11 equidistant planes that divided the optic disc diameter into 12 equal parts vertically in each eye. A reference line connecting the two termination points of the BM was drawn on each B-scan image. The distance from the reference line to the level of the anterior border of the LC was measured at three points: the maximally depressed point and two additional points (100 and 200 *μ*m from the maximally depressed point in a temporal direction). Only the temporally adjacent points were selected because the maximally depressed point was often close to the central vessel trunk, the shadow of which obscured the LC. When there was insufficient space for measuring the LCD at three points (e.g., the uppermost or lowermost B-scans or B-scans with a prominent vascular shadow), adjacent scans were used. The measurements from the 11 planes were used to calculate the mean LCD of the eye. The superior LCD was defined as the mean of 3 values obtained at the 3 uppermost B-scan images (from the 1st to the 3rd scan), the central LCD as that obtained at the 3 central-most B-scan images (from the 5th to the 7th scan), and the inferior LCD as that obtained at the 3 lowermost B-scans (from the 9th to the 11th scan).” (ii) “LCT was measured at three locations in each eye (the midhorizontal and the superior and inferior midperipheral regions of the ONH) using thin-slab maximum-intensity-projection (MIP) images.”	(i) LCT (*μ*m): overall subjects: 250.4 ± 41.6 244.9 ± 47.2 (male)/255.1 ± 37.3 (female); *p*=0.544 (ii) LCD (*μ*m): overall subjects: 425.9 ± 100.2 486.2 ± 91.4 (male)/372.0 ± 80.6 (female); *p*=0.002

Seo et al. [43]	”After the 3D image was reconstructed, seven B-scan images that divided the optic disc diameter into eight equal parts vertically were selected for each eye. These seven B-scan lines were defined as plane 1 to plane 7 (top to bottom). In this model, plane 4 corresponds to the midhorizontal plane, and planes 2 and 6 correspond to the superior and inferior midperiphery, respectively. The ALCSD was measured at each plane and defined as the distance from the Bruch's membrane opening level to the anterior LC surface.”	402.06 ± 101.46 *μ*m

Tun et al. [45]	LCD was defined as ”the distance from each anterior LC point to the peripapillary scleral (PPS) reference plane line in the central one-third of the length of BMO. The PPS reference plane was defined as a line connecting the outermost points of the anterior surface of the PPS ring. The mean depth of all LC points on the anterior LC surface was reported as the mean LC depth.”	363.65 ± 95.36 *μ*m

Thakku et al. [46]	LCD was defined as “the distance of the reconstructed anterior LC from the BMO plane. The mean depth of all points on the surface was reported as the mean LC depth. Additionally, mean depths of points along the nasal-temporal (N-T) and superior-inferior (S-I) cross sections were reported.”	403 ± 90 *μ*m

Luo et al. [22]	“Mean depth of the segmented points within the central 24 anterior scleral canal opening (ASCO) subsectors regardless of the reference plane was used for the depth measurement. Quantification of all parameters derived from the manually segmented points was performed within custom software (MATLAB version 7.3.0.267). A BMO reference plane was determined based on the 48 BMO points (2 points in each of 24 radial B-scans) as for the ASCO reference plane. Peripapillary BM and peripapillary scleral reference planes were separately defined by fitting a plane to 48 points 1700 *μ*m distal to the BMO centroid (for the BM points) and ASCO centroid (for the scleral points).”	(i) Central LD BMO (*μ*m): 402 ± 91 (ii) Central LD BM (*μ*m): 498 ± 123 (iii) Central LD ASCO (*μ*m): 309 ± 88 (iv) Central LD sclera (*μ*m): 332 ± 96

Akkaya et al. [28]	(i) ALCSD was defined as “the distance between the Bruch's membrane opening and the anterior border of the lamina cribrosa.” (ii) LCT: “anterior and posterior borders of the highly reflective region at the vertical center of the optic nerve head in horizontal SD-OCT cross sections were defined as lamina cribrosa borders, and the distance between them was defined as LCT.”	(i) LCT (*μ*m): control group: 248.50 ± 5.40 experimental group: 271.61 ± 33.96 *p* < 0.001 (ii) ALCSD (*μ*m): control group: 420.32 ± 90.26 experimental group: 351.45 ± 58.61 *p*=0.003

Eraslan et al. [29]	“LCT was measured manually on vertical lines lying between the inner and outer boundaries of the hyperreflective area temporal to the central retinal vessels. In cases where the hyporeflective image created by the nerve fibers passing through the laminar pores was too close to the temporal of the central retinal vessels in patients with thinner LCs, the measurement was performed at the points at which the inner and outer boundaries of the LC could be most clearly seen.”	Control group: 292.5 ± 33.7 *μ*m Experimental group: 209.4 ± 40.2 *μ*m *p* < 0.001

Küçük et al. [30]	(i) LCT was defined as “the distance between the LCT borders, which were the anterior and posterior borders of the highly reflective region at the vertical center of the ONH in the horizontal SD-OCT cross section.” (ii) LCD was defined as “the distance between Bruch's membrane opening and the anterior border of the LCT.”	(i) LCT (*μ*m): control group: 300.49 ± 42.6 Experiemental group (OSAS): 213.38 ± 30.7; *p* < 0.001 Mild OSAS: 223.23 ± 36.7/moderate OSAS: 219.79 ± 27.8/severe OSAS: 198.8 ± 23.1; *p*=0.068 (ii) LCD (*μ*m): control group: 411.67 ± 101.4 Experiemental group (OSAS): 397.21 ± 85.6; *p*=0.506 Mild OSAS: 408.46 ± 101.8/moderate OSAS: 404.13 ± 86.7/severe OSAS: 379.36 ± 70.2; *p*=0.639

Lee et al. [32]	“LCT was measured at three locations in each eye (the midhorizontal and the superior and inferior midperipheral regions of the ONH) using thin-slab maximum-intensity-projection (MIP) images.”	Control group: 247.95 ± 37.55 *μ*m Experimental group: 242.46 ± 31.93 *μ*m; *p*=0.616

Sirakaya et al. [33]	(i) LCT was designated as “the area between the outer and inner lines of the hyperreflective region at the vertical center of the optic nerve head; LC thickness was the perpendicular distance between those borders.” (ii) LCD was defined as ”the distance between the BMO and the anterior margin of the LC.”	(i) LCT (*μ*m): control group (group III): 279.91 ± 49.61 Experimental group (groups I and II): group I: 237.48 ± 38.53 group II: 241.42 ± 36.89; *p*=0.684 (group I versus group II); *p*=0.001 (group versus group III); *p* < 0.001 (group II versus group III) (ii) LCD (*μ*m): control group (group III): 355.34 ± 65.53 Experimental group (groups I and II): group I: 412.15 ± 58.80 Group II: 405.57 ± 55.39; *p*=0.653 (group I versus group II); *p*=0.001 (group I versus group III); *p*=0.001 (group II versus group III)

Pasaoglu et al. [47]	“LCD and LCT were measured at the 7 locations equidistant across the vertical optic disc diameter. These seven horizontal B-scan lines were defined as planes 1–7 (from superior to inferior). The average LC depth and thickness were determined as the mean values of the measurements made at seven points of the LC. ALS was defined as the anterior border of the highly reflective region beneath the internal limiting membrane at the optic disc cup on the B-scans. Distance between the reference line connecting both edges of the Bruch membrane and anterior surface of the LC at the maximally depressed point was defined as the ALS depth. The distance between the same reference line and the posterior surface of the LC again at the maximally depressed point was defined as the PLS depth. The difference between the PLS and ALS depth was taken to be the LC thickness.”	(i) ALCSD (*μ*m): control group: 359.40 ± 105.38 Experimental group: 225.00 ± 58.57 *p* < 0.01 (ii) LCT (*μ*m): control group: 210.70 ± 36.93 Experimental group: 224.75 ± 45.98 *p*=0.42

Villarruel et al. [25]	“LCD was defined as the distance between the reference line connecting both edges of the Bruch membrane (Bruch membrane opening plane) and the anterior surface of the LC. The anterior LC surface was defined as the anterior border of the highly reflective region beneath the internal limiting membrane at the optic disc cup on the B-scans. On the selected B-scans, the LC depth was measured at 3 points: the maximally depressed point and 2 additional points (100 and 200 *μ*m apart from the maximally depressed point in a temporal direction). Temporal adjacent points were selected because the maximally depressed point often was close to the central vessel trunk, which cast a shadow obscuring the LC. The average of these 3 values was defined as the LC depth of the B-scan. The LC depth of each of the B-scans was then averaged and defined as the mean LC depth for that eye.”	Control group: 387.8 ± 53.9 *μ*m Patients with IIH: 325.2 ± 92.1 *μ*m; *p*<0.01 (comparative to the control group) Patients with HTG: 493.0 ± 115.2 *μ*m; *p*<0.001 (comparative to the control group)

Demir et al. [49]	(i) ALCSD was defined as “the distance between the BMO reference plane and the anterior border of the LC.” (ii) LCT was defined as “the distance between the anterior and posterior borders of the LC. The anterior and posterior borders of the LC were defined using a highly reflective structure below the optic cup.”	(i) ALCSD (*μ*m): control group: 432.5 ± 82.1 Experimental group: 350.9 ± 70.8; *p*=0.003 (ii) LCT (*μ*m): control group: 241.3 ± 43.2 Experimental group: 166.3 ± 41.0; *p* < 0.001

Seo et al. [27]	LCD value was “determined by measuring the distance from the Bruch membrane opening (BMO) plane to the level of the anterior LC surface. The anterior surface of the LC was defined by the highly reflective structure below the optic cup. A reference line connecting the two termination points of the Bruch membrane was drawn on each B-scan image. The distance from the reference line to the level of the anterior border of the LC was then measured at three points: the maximally depressed point and two additional points located 100 *μ*m from the maximally depressed point in the temporal and nasal directions, respectively. The distance was measured on the line perpendicular to the reference line. The average value of these three points was considered as the LCD.”	Muscle-domain group: 462.79 ± 95.96 *μ*m Fat-domain group: 621.39 ± 78.39 *μ*m *p*=0.007

Moghimi et al. [50]	“The anterior and posterior borders of the highly reflective region at the vertical center of the ONH in the horizontal SD-OCT cross section were defined as the borders of the LC, and the distance between these two borders was defined as LC thickness.” (i) “Anterior laminar depth (ALD) and posterior laminar depth (PLD) were defined as the distance between the BMO and the anterior border and posterior border of the LC, respectively. The laminar thickness was defined as the distance between the anterior surface of the optic cup and the anterior border of the LC and the distance between the BMO and the internal limiting membrane (surface of the optic cup).”	(i) ALCSD (*μ*m): control group: central: 321.14 ± 106.72/superior: 324.05 ± 87.68/inferior: 280.33 ± 92.38/pool mean + SD: 308.51 ± 95.59 Experimental group: central: 330.12 ± 90.23; *p*=0.74/superior: 380.0 ± 81.51; *p*=0.04/inferior: 350.82 ± 99.73; *p*=0.05/pool mean + SD: 353.65 ± 90.49 (ii) LCT (*μ*m): control group: central: 273.3 ± 57.97/superior: 267.50 ± 93.66/inferior: 253.90 ± 59.52/pool mean + SD: 264.90 ± 70.38 Experimental group: central: 207.8 ± 47.76; *p* < 0.001/superior: 182.31 ± 48.53; *p*=0.004/inferior: 176.52 ± 42.02; *p*=0.004/pool mean + SD: 188.88 ± 46.10

Soares et al. [51]	“ALCSD was defined as the distance between the plane of Bruch's membrane opening (BMO) and the ALCS. The BMO plane was established with a line joining the limits of the BM. Three measurements of ALCS depth were made on three planes perpendicular to the BM plane: the first plane in the maximal depth of ALCS, the second plane 100 *μ*m temporal to the first plane, and the third plane 200 *μ*m temporal to the first plane. Three depth measurements were obtained, and their mean value was considered to be the final depth for each eye in both groups.”	Control group: 292.56 ± 40.71 *μ*m Experimental group: 447.96 ± 118.51 *μ*m *p*=0.001

Karaca Adıyeke et al. [52]	LCT was defined as “the distance between the anterior and posterior margins of the LC, which were determined as a highly reflective structure below the optic cup.”	Control group: 266.4 ± 10.7 *μ*m Experimental group: 285.2 ± 12.7 *μ*m (affected eye) and 283.5 ± 12.6 *μ*m (fellow eye); *p* < 0.01

Son et al. [53]	LCT was defined as “the thickness of the highly reflective region. If the lamina cribrosa margin was not defined, the auto contrast was used which was included in the program. The measurement point was the midpoint of the line connecting Bruch's membrane openings. If vascular shadows disturbed visualization of the lamina cribrosa, the measurement points were determined as centrally on the midpoint as possible where there was the least likelihood of vascular shadows.”	Control group: 260.41 ± 43.25 *μ*m Experimental group: 208.26 ± 33.36 *μ*m (affected eye of unilateral BRVO); *p* = 0.000 (comparative to the control group); 204.97 ± 37.57 *μ*m (fellow eye of unilateral BRVO); *p* = 0.000 (comparative to the control group)

Sırakaya and Bekir [54]	“Bruch's membrane opening (BMO) was defined as the distance of the line between the two endpoints of Bruch's membrane; LC is the area between the outer and inner lines of the hyperreflective region in the vertical center of the ONH.” (i) LCT was defined as “the perpendicular distance between those borders.” (ii) LCD was defined as “the distance between the BMO and anterior margin of the LC.”	(i) LCT (*μ*m): control group: 251.9 ± 37.2 Experimental group: 212.5 ± 33.3 (affected eyes); *p*<0.001 (comparative to the control group); 226.7 ± 28.8 (unaffected eyes); *p* = 0.002 (comparative to the control group) (ii) LCD (*μ*m): control group: 369.3 ± 52.3 Experimental group: 411.6 ± 71.3 (affected eyes); *p* = 0.005 (comparative to the control group); 403.31 ± 50.49 (unaffected eyes); *p* = 0.006 (comparative to the control group)

Altunel et al. [55]	“Anterior and posterior regions of the LC were defined by highly reflective structures below the ONH.” LCT was defined as “the distance between the anterior and posterior regions of the LC.”	Control group: 228.0 ± 7.1 *μ*m Experimental group: 204.4 ± 8.8 *μ*m (affected eyes); *p*<0.001 (comparative to the control group) 205.3 ± 9.3 *μ*m (fellow eyes); *p*<0.001 (comparative to the control group)

Lim et al. [56]	“LCT was measured at the vertical center of the ONH using a horizontal cross-sectional B-scan. The LCT was defined as the distance between the anterior and posterior borders of the highly reflective region. LCT was obtained from three points: the midsuperior, center, and midinferior locations. LCT was defined as the average value of the LCT at the center of the midsuperior, central, and midinferior horizontal B-scans of the ONH.”	Control group: 274.0 ± 29.4 *μ*m Experimental group: 237.0 ± 37.0 *μ*m (affected eye); *p*<0.001 (comparative to the control group) 241.4 ± 33.2 *μ*m (unafected eye); *p*<0.001 (comparative to the control group)

Akkaya and Küçük [57]	“3 frames were defined: center, midsuperior, and midinferior, which passed through the ONH, and the parameters of thickness were measured in each of these frames. During measurements of thickness, full weight to the center of the LCT plate was assigned.” LCD was defined as “the distance between the BMO and the anterior border of LCT.”	(i) LCT (*μ*m): control group: 249.1 ± 4.9 Experimental group: 174.9 ± 11.4 *p* < 0.001 (ii) LCD (*μ*m): control group: 422.0 ± 90.7 Experimental group: 403.2 ± 91.1 *p*=0.3

Lee et al. [58]	(i) LC thickness was defined as “the shortest distance between the anterior border and the posterior border of the LC. The anterior and posterior LC margins were defined by a highly reflective structure below the optic cup. Anterior and posterior LC margins were defined as the line that connected the peripheral points of the anterior and posterior LC margins. The line that connected the two termination points of Bruch's membrane was used as a baseline reference.” (ii) LC depth was measured by “calculating the average at each periphery of the anterior LC margin.”	(i) LCT (*μ*m): inferior: 218 ± 10/middle: 218 ± 10/superior: 219 ± 10/pool mean + SD: 218 ± 10 (ii) LCD (*μ*m): inferior: 426 ± 38/middle: 435 ± 41/superior: 427 ± 37/pool mean + SD: 429.33 ± 38.67

Rebolleda et al. [62]	“A reference line connecting the two Bruch's membrane limit points was drawn, and three equidistant points, corresponding to one-half and one-third of the reference, were highlighted and connected to the anterior face of the prelaminar tissue (PT) and the anterior and posterior surfaces of the LC. LCT and anterior LCD were measured at the aforementioned three points. The arithmetic mean of the three measurements was considered as the average. LCT was defined as the difference between the position of the anterior and posterior borders of the LC. LCD was determined by measuring the distance from the reference line to the level of the anterior LC surface.”	(i) ALCSD (*μ*m): control group: 347.5 ± 120.9; experimental group: 390.2 ± 120.1; *p*=0.196 (ii) LCT (*μ*m): control group: 241.2 ± 49.9; experimental group: 245.5 ± 47.4; *p*=0.720

Fard et al. [63]	“The anterior and posterior borders of the highly reflective region at the vertical center of the ONH in the horizontal SD-OCT cross section were defined as the borders of the LC, and the distance between these two borders was defined as LC thickness. Anterior laminar cribrosa depth (ALD) was measured at the anterior LC surface as the perpendicular distance from BMO distance. Anterior lamina cribrosa depth and LC thicknesses were measured at central, midsuperior, and midinferior sections of the ONH.”	(i) ALD (*μ*m): control group: central: 321 ± 107/midsuperior: 324 ± 8/midinferior: 280 ± 92/pool mean + SD: 308.33 ± 95.67; NAION: central: 339 ± 93; *p*=0.61/midsuperior: 380 ± 83; *p*=0.53/midinferior: 373 ± 133; *p*=0.30/pool mean + SD: 364 ± 103 (ii) LCT (*μ*m): control group: central: 273 ± 58/midsuperior: 268 ± 94/midinferior: 254 ± 60/pool mean + SD: 265 ± 70.67; NAION: central: 250 ± 62; *p*=0.42/midsuperior: 228 ± 52; *p*=0.17/midinferior: 235 ± 44; *p*=0.16/pool mean + SD: 237.67 ± 52.67

Lee et al. [64]	LCD was measured on “the three horizontal B-scans. A horizontal reference line was drawn by connecting the two termination points of Bruch's membrane opening (BMO) in each B-scan image. The LCD was then measured from the reference line to the level of the anterior border of the LC at the maximally depressed point and two additional points that were 100 and 200 *μ*m from the maximally depressed point in the temporal direction.”	Control group: 427.3 ± 94.1 *μ*m NAION: 390.1 ± 111.8 *μ*m NTG: 494.2 ± 92.8 *μ*m *p*=0.001

Rebolleda et al. [26]	“Reference line connecting the two ends of the BMO was defined as the BMO diameter. Three equidistant points (inferior, middle, and superior), corresponding to one-half and one-third of the reference, were highlighted and connected from this reference line to the anterior face of the prelaminar tissue (PT) and the anterior surface of the LC. Prelaminar tissue thickness (PTT) was defined as the distance between the anterior surfaces of the PT and LC. Lamina cribrosa depth (LCD) was defined as the distance from the reference line to the anterior surface of the LC. The arithmetic mean of the three measurements was registered as the average.”	Control group: 404.1 ± 70.7 *μ*m NAION: 292.1 ± 77.4 and 316.5 ± 98.5 *μ*m (unaffected fellow eye); *p* ≤0.001 (comparative to the control group)

Akkaya [73]	“Lamina cribrosa borders were defined as the posterior and anterior borders of the highly reflective area at the ONH's perpendicular center in the horizontal SD-OCT cross section.” (i) LCT was defined as “the distance between these two borders.” (ii) LCD was defined as “the distance between the BMO and the anterior border of the lamina cribrosa.”	(i) LCT (*μ*m): control group: 240.2 ± 15.8 Hyperopic nonamblyopic eyes: 251.6 ± 27.3 Amblyopic eyes: 180.9 ± 29.4 and 247.7 ± 19.0 (fellow eyes) *p* < 0.001 (amblyopia vs. control) (ii) LCD (*μ*m): control group: 391.1 ± 87.7 Hyperopic nonamblyopic eyes: 397.4 ± 75.7 Amblyopic eyes: 371.7 ± 85.8 and 272.1 ± 51.6 (fellow eyes) *p*=0.84 (amblyopia vs. control)

Lee et al. [64]	(i) LCT was measured at “3 locations in each eye (superior midperiphery, central, and inferior midperiphery regions of the ONH) using thin-slab maximum-intensity-projection (MIP) images. LCT was measured as the distance between the anterior and posterior borders at the central 3 points (with a separation of 100 mm between the points) in each MIP thin-slab image in the direction perpendicular to the anterior LC surface at the measurement point. The measurements obtained from the 3 thin-slab images were used to calculate the mean LCT of each eye.” (ii) Anterior LCD was measured in “5 horizontal B-scans (superior, superior midperiphery, center, inferior midperiphery, and inferior regions). LCD was determined by measuring the distance from the BMO plane to the level of the anterior LC surface. A reference line connecting the 2 termination points of Bruch's membrane was drawn on each B-scan image. The distance from the reference line to the level of the anterior border of the LC was measured at 3 points: the maximally depressed point and 2 additional points (100 and 200 *μ*m from the maximally depressed point in a temporal direction). The measurements from the 3 planes were used to calculate the mean LCD of the eye.”	(i) LCT (*μ*m): control group: 247.08 ± 32.70 POAG: 193.15 ± 22.19 SSOH: 260.84 ± 38.62 *p* < 0.001 (ii) ALCD (*μ*m): control group: 299.32 ± 39.78 POAG: 511.57 ± 105.36 SSOH: 421.98 ± 111.38 *p* < 0.001

Rebolleda et al. [66]	“A reference line connecting the two Bruch's membrane termination points was drawn, and three equidistant points (inferior, middle, and superior), corresponding to one-half and one-third of this reference, were highlighted and connected to the anterior face of the prelaminar tissue and anterior surface of the LC. The arithmetic mean of the three measurements (inferior, middle, and superior) was considered as the average. Lamina cribrosa depth was determined by measuring the distance from the reference line to the level of the anterior LC surface.”	400.1 ± 102.7 *μ*m

Hata et al. [67]	“The BMO was defined as the termination of the Bruch's membrane, and we measured the diameter of BMO. The BMO-anterior LC was defined as the vertical distance between the reference line connecting BMO and the anterior laminar surface.”	(i) LCD (*μ*m): CON: 376.26 ± 102.6; *p* = 0.47 (comparative to the control group)

Kim et al. [7]	“LCDs on horizontal SD-OCT B-scan images were measured at seven locations equidistant across the vertical optic disc diameter. The seven B-scan lines from the superior to the inferior regions were defined as planes 1 to 7 with plane 4 corresponding to the midhorizontal plane and planes 2 and 6 corresponding to the superior and inferior midperiphery planes, respectively. To determine the LCD, a line connecting the edges of the BMO was set as the reference plane (BMO reference line), and the LCD was measured in the direction perpendicular to the reference plane at the maximally depressed point.”	Control group: 405.88 ± 81.13 *μ*m NTG: 500.37 ± 118.97 *μ*m ADOA: 383.00 ± 85.39 *μ*m *p* < 0.001

Yang et al. [68]	“The measurement points were selected by dividing the total length of the reference line by 2 or 4 depending on the midsuperior, midinferior, or midtemporal point of the disc center. The LCD was measured from the reference line to the anterior surface of the lamina cribrosa at each point. At the same point, the LCT was defined as the minimum vertical length between the anterior and posterior surface of the prelaminar tissue and the anterior and posterior surface of the lamina cribrosa, respectively.”	(i) ALCD (*μ*m): control group: 440.9 ± 61.3; group II: 451.7 ± 74.6; group III: 455.2 ± 91.7; group IV: 464.6 ± 106.8; group V: 528.8 ± 101.8; *p*=0.001 (ii) LCT (*μ*m): control group: 212.7 ± 37.4; group II: 218.4 ± 44.0; group III: 215.0 ± 40.5; group IV: 179.2 ± 25.5; group V: 159.0 ± 14.5; *p* < 0.001

Yokota et al. [69]	“Three frames, center, midsuperior, and midinferior, which passed through the optic nerve disc were selected from these B-scans.” (i) ALD was defined as “the distance between the line connecting both ends of Bruch's membrane and the anterior border of the lamina cribrosa.” (ii) The LCT was defined as “the distance between the anterior and posterior borders of the lamina cribrosa. The anterior and posterior borders of the lamina cribrosa were defined by a highly reflective structure below the optic cup. To minimize the variation, the mean data of the three frames (center, midsuperior, and midinferior) for the ALD and the LCT analyses were considered.”	(i) ALCD (*μ*m): PDR with no-NVG: 407.0 ± 22.9; PDR with NVG: 403.9 ± 20.1; *p*=0.919 (ii) LCT (*μ*m): PDR with no-NVG: 155.0 ± 4.7; PDR with NVG: 156.9 ± 4.2; *p*=0.757

Gómez-Mariscal et al. [70]	“Three vertical and equidistant lines localized at one-half and one-third of the BMO diameter were drawn from the reference line to the anterior ONH surface and the anterior LC surface, defining the cup depth (CD) and the lamina cribrosa depth (LCD).”	Control group: 362.7 ± 98.2 *μ*m Experimental group: 369.9 ± 102.9 *μ*m *p*=0.532

LC = lamina cribrosa, LCT = lamina cribrosa thickness, cLCT = central laminacribrosa thickness, LCD = lamina cribrosa depth, ALCS = anterior lamina cribrosa surface, ALCSD = anterior lamina cribrosa surface depth, cLCSD = central anterior lamina cribrosa surface depth, BMO = Bruch's membrane opening, ONH = optic nerve head, ASCO = anterior scleral canal opening, OSAS = obstructive sleep apnea syndrome, IIH = intracranial hypertension, HTG = high-tension glaucoma, NTG = normal-tension glaucoma, BRVO = branch retinal vein occlusion, NAION = nonarteritic anterior ischaemic optic neuropathy, SSOH = superior segmental optic nerve hypoplasia, POAG = primary open-angle glaucoma, CON = compressive optic neuropathy, ADOA = autosomal dominant optic atrophy, PDR = proliferative diabetic retinopathy, and NVG = neovascular glaucoma.
